# G0S2 Promotes PD-L1 Expression in Monocytes and Influences the Efficacy of PD-1 Inhibitors in Hepatocellular Carcinoma

**DOI:** 10.3390/genes16040448

**Published:** 2025-04-13

**Authors:** Xuanshuang Du, Wenwen Zhang, Sujuan Sun, Chenghao Liu, Yuanying He, Fengling Luo, Hongyan Wu, Min Liu

**Affiliations:** 1 Department of Immunology, Taikang Medical School (School of Basic Medical Sciences), Wuhan University, Wuhan 430071, China; 2 Department of Pathology, TaiKang Medical School (School of Basic Medical Sciences), Wuhan University, Wuhan 430071, China; liuchenghao124@163.com (C.L.); 15395182380@163.com (Y.H.); 3 Hubei Key Laboratory of Tumor Microenvironment and Immunotherapy, China Three Gorges University, Yichang 443002, China; 4 College of Basic Medicine, China Three Gorges University, Yichang 443002, China

**Keywords:** G0S2, hepatocellular carcinoma, PD-L1, T cell response, PD-1 inhibitors

## Abstract

**Background:** Hepatocellular carcinoma (HCC) is a prevalent and highly lethal form of liver cancer, with limited effective treatment options, particularly in the advanced stages. Immunotherapy using PD-1 inhibitors has emerged as a promising treatment modality, yet a substantial proportion of patients exhibit resistance or fail to respond to such therapies. This study aimed to elucidate the role of G0/G1 Switch 2 (G0S2) in regulating PD-L1 expression in monocytes within the HCC tumor microenvironment and to investigate its impact on the efficacy of PD-1 inhibitors. **Methods:** Gene expression data among HCC patients treated with PD-1 inhibitors were obtained from the HCC single-cell sequencing database; immunohistochemistry was performed to detect G0S2 expression in liver cancer tissues and adjacent non-tumorous tissues of HCC patients; flow cytometry was utilized to analyze the expression of G0S2, PD-L1, CD206, and CD14 in PBMCs from HCC patients; and CD8^+^T cell proliferation and IFN-γ secretion were used to evaluate the impact of G0S2 knockdown. **Results:** Utilizing single-cell sequencing data from HCC patients, we identified that G0S2 expression was significantly elevated in the non-responders (NR) compared to responders (R) to PD-1 inhibitor therapy. The immunohistochemical analysis confirmed higher levels of G0S2 in HCC tumor tissues and adjacent non-tumorous tissues, while the flow cytometry revealed the increased expression of G0S2, PD-L1, and CD206 in peripheral blood mononuclear cells (PBMCs) from NR patients compared to R patients and healthy controls. The functional experiments involving the knockdown of G0S2 in the THP-1 monocyte cell line resulted in a significant reduction in PD-L1 expression and a concomitant increase in CD8^+^T cell proliferation and IFN-γ production. **Conclusions:** These findings indicate that G0S2 facilitates the upregulation of PD-L1 in monocytes, thereby suppressing T cell activity and contributing to resistance against PD-1 inhibitors in HCC. The high expression of G0S2 in peripheral blood monocytes offers a non-invasive and easily detectable biomarker for predicting the efficacy of PD-1 inhibitor therapy. Consequently, targeting G0S2 may enhance the responsiveness to immunotherapy in HCC patients, providing a new avenue for optimizing treatment strategies and improving patient outcomes.

## 1. Introduction

Hepatocellular carcinoma (HCC) is the most prevalent liver cancer with a high mortality rate and dismal prognosis. Although multiple treatment modalities exist for HCC, including surgical resection, loco-regional therapies, and systemic therapies, the outcomes remain suboptimal [1]. Enhancing antitumor immunity using immune checkpoint blockade (ICB), including anti-CTLA-4, anti-PD-1 (aPD1), and anti-PD-L1 (aPD-L1) antibodies, has demonstrated the potential to transform the therapeutic landscape of many cancers including HCC. Around 50% of patients with HCC receive systemic therapies, traditionally sorafenib; Lenvatinib in the first line; and pembrolizumab, regorafenib, cabozantinib or ramucirumab in the second line. One meta-analysis showed that lenvatinib determined a longer PFS and higher response rates as compared to sorafenib, although a clear survival benefit was not observed [2]. However, despite demonstrating promising a therapeutic potential across various cancer types, the efficacy of ICB in HCC patients remains inconsistent, with only a subset of patients deriving substantial benefits [3]. Identifying biomarkers associated with ICB efficacy that are also easy to detect is crucial for predicting patient responses to treatment and devising personalized therapeutic regimens. This has become a key focus in the research of immunotherapy for liver cancer.

Currently, most studies have concentrated on the expression and diversity of T cell subsets in peripheral blood, such as CD8^+^PD-1^+^T cells and tumor-specific neoantigen T cells, whose potential as biomarkers has been validated [4,5]. Monocytes, as essential components of the immune system, play pivotal roles within the tumor microenvironment (TME), particularly in regulating immune responses and facilitating tumor immune escape [6] Monocytes can differentiate into a variety of subtypes, including dendritic cells and macrophages, which control T cell activity by presenting antigens and secreting cytokines. Monocytes can differentiate into various subtypes, including macrophages and dendritic cells, participating in antigen presentation and cytokine secretion, thereby modulating T cell activity. Programmed Death-Ligand 1 (PD-L1) is a critical immunosuppressive molecule expressed on tumor cells and antigen-presenting cells. By binding to the PD-1 receptor, PD-L1 can inhibit T cell proliferation and cytotoxic functions, leading to immune escape [7,8]. Studies have found that high PD-L1 expression in monocytes is closely associated with resistance to ICB therapy, suggesting that monocytes may play a dual role in both the TME and peripheral immunity. However, the specific molecular mechanisms regulating PD-L1 expression in monocytes remain unclear and warrant further investigation. However, further research is desperately needed to determine the precise molecular pathways that control PD-L1 expression in monocytes.

Through the analysis of gene expression in monocytes from non-responders (NR) and responders (R) to PD-1 inhibitors, we identified that G0/G1 Switch Gene 2 (*G0S2*), also known as Growth Inhibition-Specific Gene 2, is significantly upregulated in the monocytes of NR patients [9]. G0S2 has garnered extensive attention in recent years as a regulatory factor, initially named for its role in the G0/G1 cell cycle transition. The protein encoded by the *G0S2* gene is involved in various biological processes, including cell cycle regulation, lipid metabolism, and immune modulation. Specifically, G0S2 interacts with Adipose Triglyceride Lipase, inhibiting the mobilization and oxidation of fatty acids, thereby maintaining lipid metabolic balance. Additionally, G0S2 exhibits diverse regulatory functions in apoptosis, autophagy, and energy metabolism. In the context of tumor biology, the mechanisms by which G0S2 operates are not yet fully understood. Existing studies suggest that G0S2 may have dual roles in multiple cancer types, potentially acting as a tumor suppressor by regulating the cell cycle and apoptosis, while also supporting tumor cell growth and survival through metabolic pathway modulation. Particularly in HCC, the expression levels and regulatory mechanisms of G0S2 have not been thoroughly investigated, and its potential role in immune regulation remains unreported.

This study aims to validate the regulatory role of G0S2 in promoting PD-L1 expression in monocytes, elucidate its impact on T cell function within the immunosuppressive environment, and further explore its potential as a predictive biomarker and therapeutic target for ICB efficacy. Our findings are expected to provide new insights and possible combination therapy strategies to enhance the ICB efficacy in HCC patients.

## 2. Materials and Methods

### 2.1. Exploration of the Relationship Between PD-1 Inhibitor Resistance and Gene Expression in Peripheral Blood Mononuclear Cells

Gene expression data for NR and R among HCC patients treated with PD-1 inhibitors were obtained from the HCC single-cell sequencing database (HRA004885). The patients were derived from the KEYNOTE394 trial (ClinicalTrials.gov ID: NCT03062358) [10], in which patients with advanced HCC received pembrolizumab via intravenous infusion on Day 1 of each 3-week cycle for up to 35 cycles, in addition to best supportive care. Patients were initially categorized into R and NR based on their treatment responses. Seven blood samples were collected prior to pembrolizumab treatment (4 from the NR group and 3 from the R group) [11]. Data preprocessing was performed using the Seurat package (v4.0.3), including data normalization, identification of highly variable genes, and data scaling. Principal Component Analysis (PCA) was conducted for dimensionality reduction, followed by visualization clustering using UMAP or t-SNE methods. Differentially expressed genes between NR and R patients were identified using the FindMarkers function, with selection criteria set at |log_2_FoldChange| > 0.25 and adjusted *p*-value < 0.05. Volcano plots were generated using the ggplot2 package to highlight differentially expressed genes, with significantly upregulated and downregulated genes marked in different colors.

### 2.2. Validation of G0S2 Expression Using a Validation Dataset

**Figure 1:**
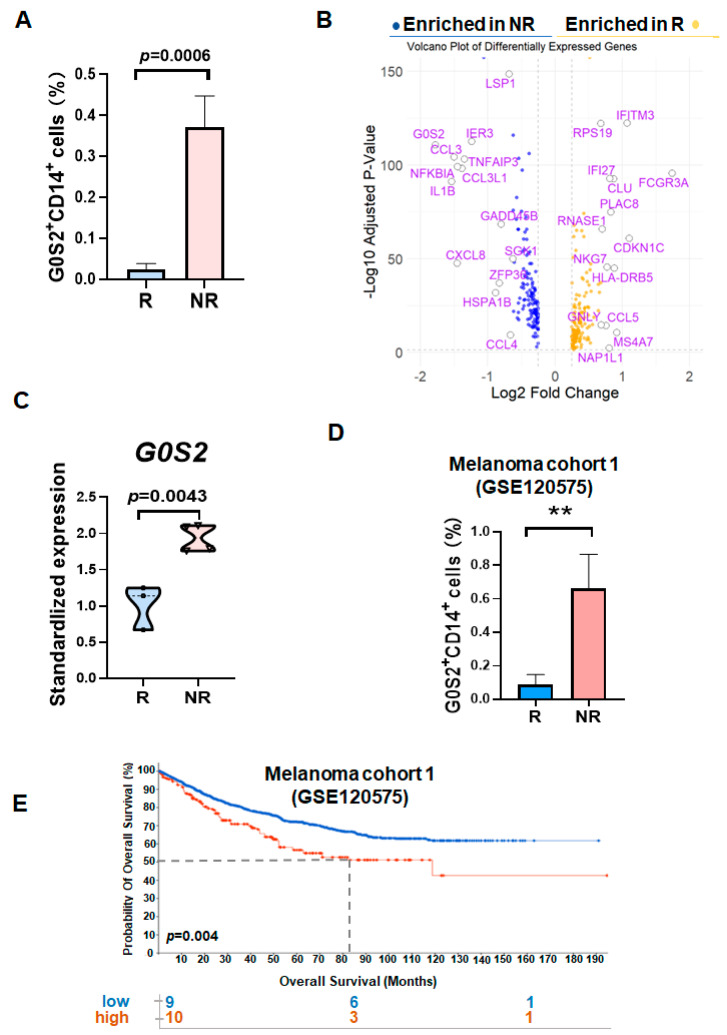
High expression of G0S2^+^CD14^+^ monocytes in HCC patients non-responsive to PD-1 inhi-itors. (**A**) Volcano plot illustrating gene expression in monocytes with an absolute log₂FoldChange > 0.25. (**B**) R analysis of standardized G0S2 expression levels in monocytes of non-responders and responders. (**C**) R analysis of the positive expression rate of G0S2^+^CD14^+^ cells in the peripheral blood of non-responders and responders. (**D**) R analysis validating the positive expression rate of G0S2^+^CD14^+^ cells in PBMCs of non-responders and responders within the melanoma dataset (GSE120575). (**E**) R analysis of survival rates between high and low G0S2^+^CD14^+^ cell expression groups in GSE120575. ** *p* < 0.01.

To validate the differential expression of G0S2 in tumor patients, the melanoma validation dataset (GSE120575) from the GEO database was analyzed. The limma package was used to process the validation dataset, including data import, background correction, and normalization. Gene expression data for NR and R patients were extracted, and the differential expression of G0S2 between these two groups was calculated. Box plots were created using the ggplot2 package to display the differences in G0S2 expression levels between NR and R patients, and statistical significance was assessed using tests such as the t-test or Mann–Whitney U test. To further validate the correlation between G0S2 expression and survival prognosis, survival analysis was conducted using the survival and survminer packages. Kaplan–Meier survival curves were added to Figure 1 to compare the survival rates of patients with high and low G0S2 expression, with p-values and hazard ratios (HR) calculated for survival differences, and confidence intervals annotated on the survival curves.

### 2.3. Patient Sample Collection

Blood samples from HCC patients were obtained from the Oncology Department of Renmin Hospital of Wuhan University, tissue sections were sourced from the Pathology Department of Renmin Hospital of Wuhan University, and samples from healthy volunteers were collected from the Health Examination Center of Renmin Hospital of Wuhan University (patient information in Supplementary Table S2). All participants provided informed consent, and the study was approved by the Ethics Committee of the Life Sciences Medical Ethics Committee of Wuhan University (Approval No. 20220007). Collected peripheral blood samples were immediately stored in EDTA anticoagulant tubes and peripheral blood mononuclear cells (PBMCs) were isolated within 2 h using the Ficoll-Paque density gradient centrifugation method (Catalog No. 17-1440-02, GE Healthcare, Chicago, IL, USA) Blood samples were diluted 1:1 with PBS and gently layered over Ficoll solution, followed by centrifugation at 800× *g* for 30 min without braking. The PBMC layer was collected, washed twice with PBS, and stored for subsequent experiments.

### 2.4. Multiplex Immunohistochemical Staining (mIHC)

We performed mIHC staining on tissues using an Opal 7-Color IHC Kit (Catalog No. NEL811001KT, Akoya, Waltham, MA, USA). The stained slides were scanned using a Vectra 3.0 multispectral imaging system (Akoya, Waltham, MA, USA). The immunofluorescence markers used were CD14 (Catalog No. 75181T, Cell Signaling Technology, Boston, MA, USA), CD68 (Catalog No. M087601-2CN, Agilent Technologies, Santa Clara, CA, USA), and G0S2 (Catalog No.12091-1-AP, Proteintech, Chicago, IL, USA). The effects of each primary antibody were visualized via tyramide signal amplification in combination with specific fluorochromes from multiplex immunohistochemistry kits. We strictly adhered to the instructions provided by the manufacturer throughout the entire mIHC procedure. mIHC-stained slides were scanned under fluorescence illumination using a Vectra 3.0 multispectral microscope system and InForm 3.0 software. On each slide, Vectra automatically collects fluorescence spectra from 420 nm to 720 nm with an optimal exposure time interval of 20 nm. Next, the acquired images are combined into a single stacked image, preserving all IF-labeled particle spectral signatures.

### 2.5. Flow Cytometry Staining

Flow cytometry was utilized to analyze the expression of G0S2, PD-L1, CD206, and CD14 in PBMCs from HCC patients. PBMCs were resuspended at a concentration of 1 × 10^6^ cells per tube in flow cytometry buffer and incubated with FcγR blocking reagent (Catalog No. 422302, BioLegend, San Diego, CA, USA) at 4 °C for 15 min to reduce non-specific binding. Subsequently, cell membrane staining was performed by adding anti-PD-L1-PE (BioLegend, Catalog No. 329706), anti-CD206-FITC (Catalog No. 321106, BioLegend), and anti-CD14-APC (Catalog No. 325608, BioLegend) antibodies, followed by incubation at 4 °C in the dark for 30 min. To detect intracellular G0S2 expression, cells underwent fixation and permeabilization using the BD Cytofix/Cytoperm kit (Catalog No. 554714, BD Biosciences, San Jose, CA, USA) after membrane staining. Following fixation and permeabilization, cells were incubated with primary anti-G0S2 antibody (Catalog No. G0S2-101AP, Thermo Fisher Scientific, Waltham, MA, USA) at 4 °C in the dark for 30 min. After washing, cells were incubated with PE-CY5-conjugated secondary antibody (Catalog No. 405207, BioLegend) at 4 °C in the dark for 30 min. Post-staining, cells were washed and resuspended in 500 μL of flow cytometry buffer. Samples were analyzed using the BD LSRFortessa flow cytometer, and data were processed with FlowJo software 10.8.1. Quantitative analysis of G0S2, PD-L1, CD206, and CD14 expression was performed, and cell subpopulations were categorized based on marker expression levels.

### 2.6. RT-PCR

Reverse transcription-polymerase chain reaction (RT-PCR) was employed to assess the expression levels of *G0S2* and *PD-L1 (CD274)* in different groups of THP-1 cells. Total RNA was extracted using the Trizol method, reverse transcribed into cDNA, and subjected to quantitative PCR using the SYBR Green kit (Catalog No. RR820A, Takara, Japan). Gene expression was quantified using the delta–delta CT method (2^−(Ct_Target gene − Ct_GAPDH)^). The Trizol reagent was purchased from Wuhan Huiyu Biotechnology Co., Ltd. (Catalog No. RHYC01, Wuhan, China), the reverse transcription kit from Wuhan Saiwei Biotechnology Co., Ltd. (Catalog No. G3332-50, Wuhan, China), and the SYBR Green fluorescent dye from TOLOBIO (Catalog No. 22204-01, Wuxi, China). Human G0S2 and PD-L1 primers were synthesized by Wuhan Qingke Biotechnology Co., Ltd. (Wuhan, China). Primer sequences were as follows: *G0S2*-F: AGCAGTTGGTGACCATGTCG, *G0S2*-R: TGGAGATCTCCTGCTTGAGG; *CD274*-F: CCTTCCGTGTTCCTACCC, *CD274*-R: GCCTGCTTCACCACCTTC; *GAPDH*-F: ACCACAGTCCATGCCATC, *GAPDH*-R: TCCACCACCCTGTTGCTG.

### 2.7. Western Blot Analysis

Cells were harvested, and proteins were lysed using RIPA buffer. Proteins were separated by SDS-PAGE electrophoresis and transferred to PVDF membranes. Membranes were probed with primary antibodies against G0S2 (Catalog No. G0S2-101AP, Thermo Fisher Scientific, Waltham, MA, USA), PD-L1 (Catalog No. 66248-1-Ig Proteintech), and GAPDH (Catalog No. 60004-1-Ig, Proteintech). After incubation with secondary antibodies, signals were detected using the chemiluminescence method (ECL). Reagents used included RIPA lysis buffer (PC101), protease inhibitor (GRF101), phosphatase inhibitor (GRF102), 5× loading buffer (LT101), rapid preparation kit for 7.5% PAGE gel (PG211), protein marker (WJ102), non-protein blocking solution (PS108P), and ECL substrate (SQ202L), all purchased from Yease Company(Wuhan, China).

### 2.8. T Cell Proliferation and IFN-γ Detection

To evaluate the impact of G0S2 knockdown on CD8^+^T cell proliferation and IFN-γ secretion, G0S2-silenced THP-1 cells were co-cultured with CD8^+^T cells from healthy donors. The experimental procedure was as follows: Specific siRNA was used to knock down G0S2 expression in THP-1 cells (using Lipofectamine RNAiMAX reagent, Catalog No. 13778075, Invitrogen, Carlsbad, CA, USA). After 24 h of transfection, cells were collected and washed to remove transfection reagents. PBMCs were isolated from the peripheral blood of healthy donors using the Ficoll-Paque density gradient centrifugation method (Catalog No. 17-1440-02, GE Healthcare, Little Chalfont, Buckinghamshire, UK). CD8 ^+^ T cells were separated using negative selection with a CD8^+^T cell isolation kit (Catalog No. 130-045-201, Miltenyi Biotec, Bergisch Gladbach, Germany). G0S2-knockdown THP-1 cells were mixed with CD8 + T cells at a 1:1 ratio and added to a co-culture system containing complete RPMI 1640 medium (Catalog No. 11875-093, Gibco, Grand Island, NY, USA). Cells were incubated at 37 °C with 5% CO_2_ for 48 h. After 48 h of co-culture, cells were stimulated with medium containing PMA (50 ng/mL) and ionomycin (1 μg/mL) for 4 h (Catalog Nos. P8139 and I9657, Sigma-Aldrich, St. Louis, MO, USA), along with protein transport inhibitors such as Brefeldin A (Catalog No. 420601, BioLegend). Cells were collected and washed with flow cytometry buffer. Fcγ receptor blocking reagent (Catalog No. 422302, BioLegend) was added and incubated at 4 °C for 10 min to reduce non-specific binding. Anti-CD8-APC (Catalog No. 301014, BioLegend) was added for cell surface staining, followed by incubation at 4 °C in the dark for 30 min. Cells were fixed and permeabilized using the BD Cytofix/Cytoperm kit (Catalog No. 554714, BD Biosciences). Anti-IFN-γ-PE antibody (Catalog No. 506506, BioLegend) was added and incubated at 4 °C in the dark for 30 min. Cells were washed and resuspended in 500 μL flow cytometry buffer. Detection was performed using the BD LSRFortessa flow cytometer, and data were analyzed using FlowJo software to quantify the percentage of CD8^+^ IFN-γ^+^ T cells, assessing the impact of G0S2 knockdown on CD8^+^T cell function.

CD8^+^T cells were labeled with CFSE (5-(6)-Carboxyfluorescein diacetate succinimidyl ester, Catalog No. C34554, Invitrogen) to evaluate cell proliferation. G0S2-knockdown THP-1 cells were mixed with CFSE-labeled CD8^+^T cells at a 1:1 ratio and added to a co-culture system containing complete RPMI 1640 medium. Cells were incubated at 37 °C with 5% CO_2_ for 48 h. CFSE fluorescence intensity was detected using the BD LSRFortessa flow cytometer to assess T cell proliferation. FlowJo software was used to analyze flow cytometry data, calculating the percentage of proliferating CD8^+^T cells and the average number of cell divisions.

### 2.9. Data Statistical Analysis

All experiments were performed at least three times, and data are presented as mean ± standard deviation (mean ± SD). Differences between groups were analyzed using one-way ANOVA or two-way ANOVA with GraphPad Prism 9 software (GraphPad Software). A *p*-value of less than 0.05 (*p* < 0.05) was considered statistically significant.

## 3. Results

### 3.1. High Expression of G0S2 in Peripheral Blood Mononuclear Cells of HCC Patients Is Closely Associated with PD-1 Resistance

We analyzed the HCC single-cell dataset HRA004885. Volcano plots were generated to display genes with an absolute log_2_FoldChange > 0.25, highlighting the top ten genes ranked by the combined log_2_FoldChange and *p*-value (Figure 1A). We focused on the *G0S2* gene, which was the top-ranked gene overall. Further analysis of the standardized expression levels of G0S2 in PBMCs revealed that the G0S2 expression was significantly higher in the NR patients compared to the R patients (Figure 1B). *G0S2* is an important gene involved in various physiological and pathological processes. It can influence the activation state and immune functions of monocytes and macrophages by regulating lipid metabolism, apoptosis, and inflammatory responses. This has led to increasing attention to its role in the cancer immune environment. The analysis of the positive expression rate of the G0S2^+^CD14^+^cells showed that the proportion of G0S2^+^CD14^+^ cells in the NR patients was significantly higher than that in the R patients (Figure 1C). To validate the broader applicability of this finding, we conducted further validation using a melanoma patient dataset (GSE120575). The results demonstrated that the proportion of G0S2^+^CD14^+^ cells was also significantly higher in the NR patients compared to that in the R patients in this dataset (Figure 1D). The survival analysis further indicated that the high expression of G0S2^+^CD14^+^ cells was significantly associated with poorer patient survival rates (Figure 1E), suggesting that G0S2 in peripheral blood mononuclear cells may serve as an important biomarker for predicting the efficacy of PD-1 inhibitor therapy. Taken together, these results suggest that high expression of G0S2 in monocytes may play a critical role in PD-1 inhibitor resistance by affecting immunosuppressive pathways.

### 3.2. High Expression of G0S2 in Monocytes and HCC Tissues of HCC Patients

**Figure 2:**
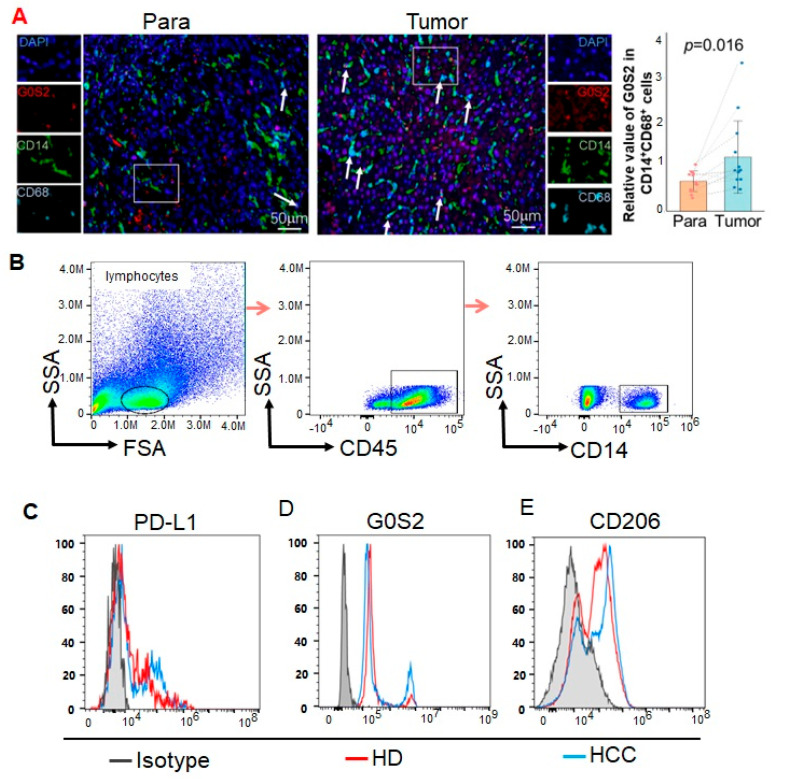
Expression of G0S2 in monocytes and HCC tissues of HCC patients. (**A**) G0S2 expression in CD14^+^CD68^+^ cells from HCC tumor vs. para-tumor tissues, as determined by multicolor immunohistochemistry. Bar plot with mean ± SD for tumor (blue) and para-tumor (orange) tissues, overlaid with individual paired data points (*n* = 13). Dashed gray lines connect each patient’s tumor and para-tumor values. (**B**–**E**) Flow cytometry analysis of PD-L1, G0S2, and CD206 expression in monocytes of PBMCs from HCC patients and healthy individuals. ns: not significant.

To investigate the expression of G0S2 in liver cancer tissues, we performed mIHC using antibodies against CD14, CD68, and G0S2 on an HCC tissue microarray to enable double staining with the monocyte markers. Overall, G0S2 expression is significantly higher in tumor tissues compared to para-tumor tissues (Figure 2A, *p* = 0.016). To further quantify this difference, we calculated the fold change in G0S2 expression (Tumor/Para) for each patient pair (Supplementary Figure S1). We set a 1.5-fold increase as the threshold for a marked elevation in expression. Our data show that approximately 62% of the patient pairs (8 out of 13) exhibit at least a 1.5-fold increase in G0S2 expression in tumor tissues relative to the corresponding para-tumor tissues. This elevated expression in the tumor tissues suggests that higher G0S2 levels may correlate with reduced sensitivity to PD-1 therapy, considering that PD-1 antibodies achieve response rates of around 30–40% in HCC.

To explore the expression of G0S2 in peripheral blood mononuclear cells of HCC patients, we conducted flow cytometry analysis on PBMCs from HCC patients and healthy controls from Renmin Hospital of Wuhan University. The cell population markers confirmed the successful isolation of the CD14^+^ monocyte population (Figure 2B). The flow cytometry results revealed that, compared to the healthy controls, the expression of PD-L1 in the monocytes of the HCC patients was significantly upregulated (Figure 2C), indicating that PD-L1 may play a crucial role in HCC immune escape. Additionally, the G0S2 expression in the monocytes of the HCC patients was also significantly upregulated compared to that of the healthy controls (Figure 2D). We also examined the expression levels of the M2-type marker CD206 in monocytes from the HCC patients and healthy individuals. The results showed a significant increase in CD206 expression in the monocytes of the HCC patients (Figure 2E), suggesting that these cells may possess immunosuppressive characteristics that contribute to tumor immune escape.

### 3.3. G0S2 Promotes PD-L1 Expression in Monocytes

**Figure 3:**
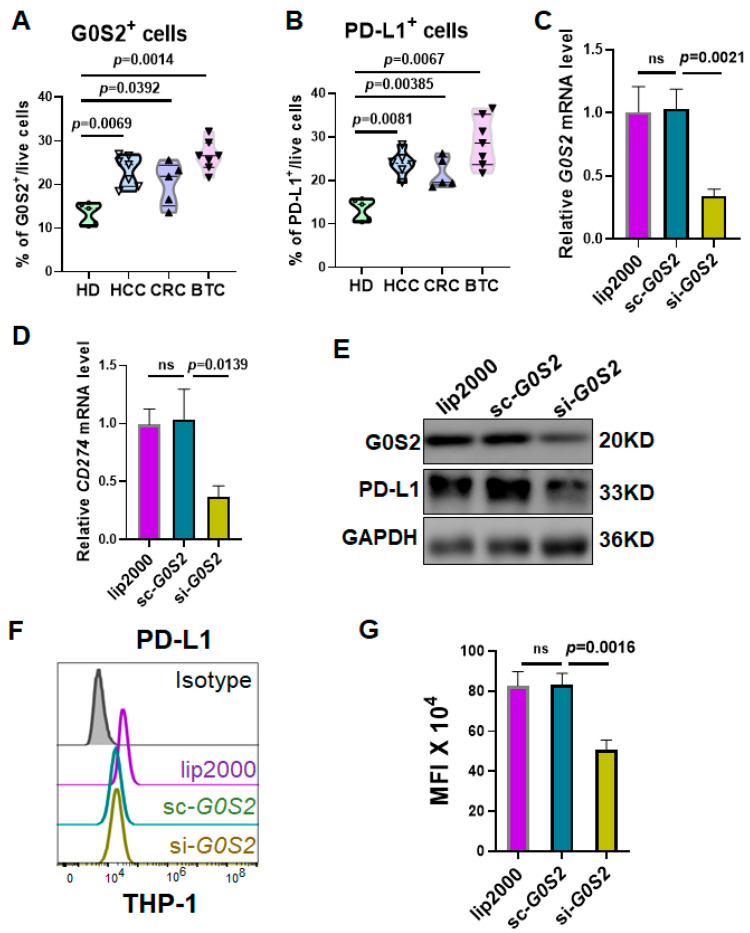
G0S2 enhances PD-L1 expression in monocytes. (**A**,**B**) R analysis of the proportion of G0S2^+^ and PD-L1^+^ cells in peripheral blood mononuclear cells of healthy donors and patients with HCC, CRC, and BTC in the HRA004885 dataset. (**C**) RT-PCR validation of G0S2 knockdown in THP-1 cells using specific siRNA. (**D**) RT-PCR validation of PD-L1 expression after G0S2 knockdown in THP-1 cells. (**E**) Western Blot analysis of PD-L1 expression in THP-1 cells following G0S2 knockdown. (**F**,**G**) Flow cytometry analysis of PD-L1 expression in THP-1 cells after G0S2 knockdown. ns: not significant.

High PD-L1 expression is closely associated with poor outcomes of ICB therapy [12]. Although previous studies have demonstrated PD-L1 expression in various tumors and immune cells, the regulatory mechanisms of PD-L1 expression in monocytes remain insufficiently elucidated. *G0S2*, a gene related to cell proliferation and metabolism [13], has gained attention in recent years, yet its role in immune cells has been minimally studied. To investigate the regulatory effect of G0S2 on PD-L1 expression in monocytes, we first analyzed the expression levels of G0S2 and PD-L1 in the HCC single-cell dataset HRA004885. Figure 3A,B show that the proportion of G0S2^+^ and PD-L1^+^ cells in the peripheral blood mononuclear cells of the HCC patients was significantly higher than that in the healthy individuals, and similar proportions were observed in the patients with colorectal cancer (CRC) and biliary tract cancer (BTC) (Figure 3A,B). This suggests that G0S2 may participate in immune regulation and ICB resistance in HCC patients by upregulating PD-L1 expression. To further validate the regulatory role of G0S2 on PD-L1 expression, we employed specific siRNA to knock down G0S2 expression in the THP-1 monocyte cell line and assessed PD-L1 expression changes using RT-PCR and Western Blot. The results demonstrated that the successful knockdown of G0S2 (Figure 3C,E) significantly reduced PD-L1 mRNA and protein expression (Figure 3D,E). Furthermore, the flow cytometry analysis confirmed that knocking down G0S2 significantly decreased the proportion of PD-L1^+^ cells in the THP-1 cells (Figure 3F,G), indicating that G0S2 may promote an immunosuppressive state by upregulating PD-L1 expression in monocytes. Collectively, these results suggest that G0S2 regulates PD-L1 expression in monocytes, potentially playing a key role in immune escape and PD-1 inhibitor resistance in HCC.

### 3.4. G0S2 Expression in Monocytes Inhibits T Cell Activity

**Figure 4:**
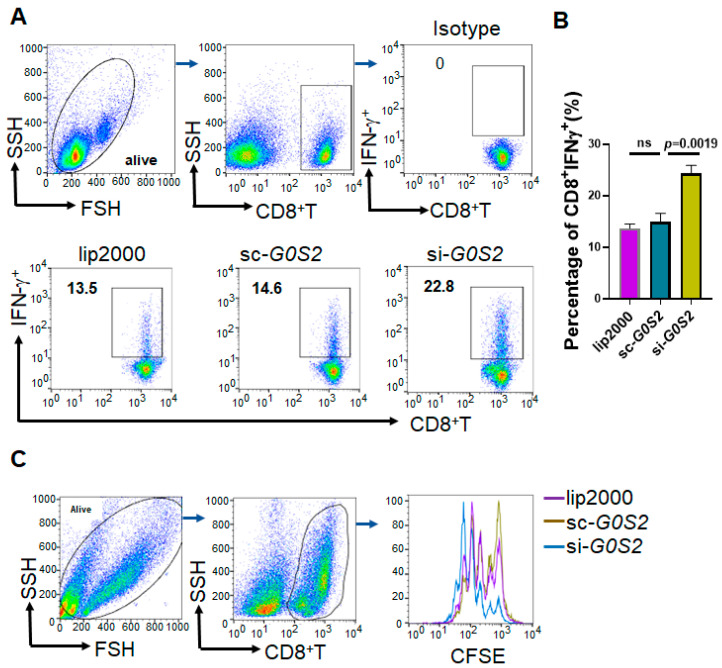
G0S2 expression in monocytes inhibits T cell activity. (**A**,**B**) Flow cytometry analysis of IFN-γ production in CD8^+^T cells co-cultured with G0S2-knockdown THP-1 cells. (**C**) Flow cytometry analysis of CD8^+^T cell proliferation after co-culture with CFSE-labeled CD8^+^ T cells and G0S2-knockdown THP-1 cells. ns: not significant.

To investigate the impact of G0S2 expression in monocytes on T cell function, we conducted experiments where G0S2 was knocked down in THP-1 cells and co-cultured with human CD8^+^T cells. The flow cytometry analysis revealed that G0S2-knockdown THP-1 cells significantly increased IFN-γ production in the CD8^+^ T cells after co-culture (Figure 4A,B), indicating enhanced T cell activity due to G0S2 knockdown. Specifically, compared to the control groups (lip2000 and sc-G0S2), the proportion of CD8^+^ IFN-γ^+^ T cells was significantly elevated in the si-G0S2 group. To further assess the effect of G0S2 knockdown on T cell proliferation, THP-1 cells were co-cultured with CFSE-labeled human CD8^+^T cells, followed by flow cytometry analysis. The results showed that the G0S2-knockdown THP-1 cells significantly promoted CD8^+^T cell proliferation (Figure 4C). These findings suggest that G0S2 in monocytes may inhibit T cell activity by regulating PD-L1 expression, and that knocking down G0S2 can enhance the antitumor functions of T cells.

To further elucidate the mechanism of the interaction between monocytes and T cells, we performed additional co-culture experiments using a transwell system (Supplementary Figure S2). When G0S2-knockdown THP-1 cells and CD8⁺ T cells were separated by a permeable membrane, no significant change in T cell function (e.g., IFN-γ production) was observed compared to the control. This indicates that the enhanced T cell activity observed in our direct co-culture experiments is predominantly dependent on direct cell-to-cell contact between monocytes and T cells, rather than on soluble factors alone.

## 4. Discussion

Our results demonstrate that G0S2 expression is significantly elevated in both tumor tissues and peripheral blood mononuclear cells of HCC patients. Importantly, G0S2 knockdown in THP-1 cells resulted in a marked reduction in PD-L1 expression, as confirmed. Furthermore, co-culture experiments with human CD8⁺ T cells revealed that G0S2-knockdown monocytes led to enhanced T cell proliferation and increased IFN-γ production, indicating that the immunosuppressive function of monocytes is at least partly mediated through G0S2-dependent PD-L1 regulation. These comprehensive data clearly support the conclusion that G0S2 is a key regulator of PD-L1 expression in monocytes, making it a promising predictive biomarker and potential therapeutic target in HCC immunotherapy.

As a growth inhibition-specific gene, *G0S2* has been shown to participate in cell growth and metabolic regulation across various cell types [14]. However, its function in immune cells has not been extensively studied. Monocytes are a vital type of immune cell in the blood, capable of migrating into the TME and differentiating into TAMs [15]. TAMs exert immunosuppressive effects within the tumor microenvironment, playing a critical role in tumor immune escape [16]. PD-L1 is a key factor in tumor immune escape, inhibiting T cell activation and proliferation by binding to the PD-1 receptor on T cells, thereby allowing tumor cells to evade immune surveillance [17,18]. In our study, we observed that the high expression of G0S2 in the PBMCs of HCC patients was significantly correlated with the upregulation of PD-L1. This suggests that G0S2 may play a pivotal role in tumor immune escape by regulating TAMs. An immunohistochemical analysis demonstrated that G0S2 expression was significantly higher in the tumor tissues compared to the adjacent non-tumorous tissues, further confirming its important role within the tumor microenvironment. The flow cytometry results also confirmed that the G0S2 expression in the PBMCs of HCC patients was significantly higher than that in the healthy controls, positively correlating with the expression of immunosuppressive molecules such as PD-L1 and CD206. This indicates that these immune cells possess immune-escaping characteristics, thereby promoting tumor growth and metastasis. The high expression of G0S2 in monocytes may thus be a key factor in tumor immune escape and resistance to PD-1 inhibitors.

To further explore the mechanism by which G0S2 regulates PD-L1 expression in monocytes, we conducted G0S2 knockdown experiments in the THP-1 cell line. The results showed that knocking down G0S2 significantly reduced PD-L1 expression at both the mRNA and protein levels. Additionally, co-culture with human CD8⁺ T cells revealed that the G0S2-knockdown THP-1 cells significantly enhanced IFN-γ production and the proliferation of T cells. This indicates that high expression of G0S2 inhibits the antitumor activity of T cells through PD-L1 regulation. These findings suggest that G0S2 promotes tumor immune escape by suppressing T cell function through PD-L1 expression regulation. This aligns with existing research indicating that monocytes and macrophages play significant roles in immune regulation within the tumor microenvironment, impacting the effectiveness of immunotherapy [19,20]. Our study provides a new perspective for understanding the role of monocytes in immune escape.

Furthermore, our experimental results indicated that the expression of the M2-type marker CD206 decreased following G0S2 knockdown [21], suggesting that G0S2 may play an important role in the differentiation and function of M2-type monocytes. M2-type monocytes and macrophages are generally considered to be immunosuppressive and tumor-promoting cells within tumors. High expression of G0S2 in these cells may help maintain their immunosuppressive functions, thereby further supporting tumor growth and dissemination.

G0S2 is highly expressed in HCC patients and is associated with PD-L1 expression and the suppression of CD8^+^ T cell function. Recent studies have highlighted that the use of predictive biomarkers in the adjuvant setting can significantly improve patient outcomes by guiding treatment intensification or modification. It is reported that immune-related lncRNA signature possesses promising prognostic value in HCC and may have the potentiality to predict clinical outcome of ICB immunotherapy [22]. RPS3A may serve as a therapeutic target in and predict the efficacy of ICB therapy for HCC [23]. Our data suggest that quantifying G0S2 expression in PBMCs may help identify HCC patients who are less likely to respond to standard PD-1 inhibitor therapy. These patients could be considered for alternative or combination adjuvant strategies, such as incorporating G0S2-targeted agents, combining PD-1 inhibitors with other immunomodulatory approaches (e.g., CTLA-4 blockade), or even integrating interventions that modulate the gut microbiome, such as fecal microbiota transplantation (FMT). By implementing routine G0S2 monitoring, clinicians may be able to detect early signs of immunotherapy resistance and adjust adjuvant treatment plans accordingly, thereby reducing recurrence risk and improving overall survival. Future prospective studies are needed to validate G0S2 as a reliable adjuvant biomarker and to integrate it into multi-modal treatment algorithms.

Our findings suggest that G0S2 upregulation in monocytes, which leads to increased PD-L1 expression, is particularly relevant for predicting the efficacy of PD-1/PD-L1 blockade therapies, such as pembrolizumab, nivolumab, and camrelizumab. There are a lot of ongoing or completed clinical trials focused on PD-1/PD-L1 blockade therapies for HCC (Supplemental Table S3). Although our study focused on anti-PD-1 monotherapy, recent meta-analyses [24,25] have demonstrated that regorafenib is more effective than other treatments (cabozantinib, nivolumab, or placebo) for patients with HCC who have not responded to initial sorafenib therapy. It is important to note that regorafenib primarily functions as a multi-kinase inhibitor and does not directly target the PD-1/PD-L1 axis. However, as in the immunotherapy strategies evolve, combination regimens involving PD-1/PD-L1 inhibitors and agents like regorafenib are being investigated. In such combinations, our data raise the possibility that G0S2 expression could also serve as a predictive biomarker, guiding treatment decisions and helping to identify patients who might benefit from the addition of PD-1/PD-L1 blockade to regorafenib therapy. Future studies will be needed to explore whether G0S2 detection can predict responses in these combination settings, thereby further refining therapeutic strategies for HCC.

Fecal microbiota transplantation (FMT) has been shown to improve the efficacy of PD-1 inhibitors by restoring a balanced gut microbial ecosystem, which in turn can enhance systemic antitumor immunity [26,27]. By normalizing the gut–liver axis, FMT may help re-establish effective antitumor immunity, making subsequent PD-1 blockade therapy more effective. Our future work may focus on investigating how FMT affects the expression of G0S2 and PD-L1 in the PBMCs of HCC patients. By elucidating the underlying mechanisms through which FMT alters these molecular pathways, we aim to uncover potential strategies to reduce PD-1 resistance and further improve the therapeutic outcomes of immunotherapy in HCC.

Despite uncovering the role of G0S2 in regulating PD-L1 expression and affecting T cell function in HCC patients, several questions remain to be addressed. Firstly, the specific molecular mechanisms by which G0S2 regulates PD-L1 expression need to be further explored. Future studies could employ transcription factor binding site analysis and gene knockout experiments to verify the upstream regulatory mechanisms of G0S2 in PD-L1 regulation. Secondly, it is necessary to validate whether G0S2 exhibits similar functions and its generalizability in immunosuppression across different cancer types. Additionally, investigating whether G0S2 is associated with the expression and function of other immune checkpoint molecules is an important direction for future research. Existing studies have shown that G0S2 can regulate lipid metabolism and apoptosis, which may influence tumor cells and immune cells through different mechanisms [7,8,9,11,12,13,]. We hypothesize that G0S2 may promote PD-L1 expression by regulating the metabolic state of cells, thereby inhibiting T cell activity. The high expression of G0S2 in monocytes may induce PD-L1 upregulation through multiple signaling pathways, including NF-κB and STAT3 pathways [28,29], playing a crucial role in the formation of an immunosuppressive microenvironment. Additionally, using *G0S2*-deficient mice for in vivo animal studies could further validate the impact of G0S2-mediated PD-L1 regulation on the efficacy of ICB therapy.

This study demonstrates elevated G0S2 expression in HCC patients and its role in inhibiting CD8^+^T cell function by promoting PD-L1 expression, potentially leading to resistance to PD-1 inhibitors. Our results suggest that G0S2 could serve as an important biomarker for predicting the efficacy of PD-1 inhibitors in HCC patients and provide a theoretical basis for developing combination therapy strategies targeting G0S2. Through further mechanistic studies and clinical validation, G0S2 has the potential to become a key regulatory factor in improving the effectiveness of immunotherapy in HCC patients. Our research lays the groundwork for exploring G0S2 as a new immunotherapy target, opening new avenues for immunotherapeutic strategies in HCC and other cancers.

## 5. Conclusions

The findings indicate that G0S2 facilitates the upregulation of PD-L1 in monocytes, thereby suppressing T cell activity and contributing to resistance against PD-1 inhibitors in HCC. The high expression of G0S2 in peripheral blood monocytes offers a non-invasive and easily detectable biomarker for predicting the efficacy of PD-1 inhibitor therapy. Consequently, targeting G0S2 may enhance the responsiveness to immunotherapy in HCC patients, providing a new avenue for optimizing treatment strategies and improving patient outcomes.

## 6. Patents

Blood samples from HCC patients were obtained from the Oncology Department of Renmin Hospital of Wuhan University, tissue sections were sourced from the Pathology Department of Renmin Hospital of Wuhan University, and samples from healthy volunteers were collected from the Health Examination Center of Renmin Hospital of Wuhan University. All participants provided informed consent, and the study was approved by the Ethics Committee of the Life Sciences Medical Ethics Committee of Wuhan University (Approval No. 20220007).

## Data Availability

HCC single-cell sequencing database (HRA004885) and the melanoma validation dataset (GSE120575) from the GEO database.

## Supplementary Materials

The following supporting information can be downloaded at https://www.mdpi.com/article/10.3390/genes16040448/s1: Figure S1: Related to Figure 2. The fold change in G0S2 expression (Tumor/Para) for each patient pair; Figure S2: Related to Figure 4. Flow cytometry analysis of IFN-γ production in CD8^+^T cells co-cultured with G0S2-knockdown THP-1 cells using a transwell system. Supplementary Table S1. Demographic and clinical characteristics of individuals enrolled in this study. Supplementary Table S2. Demographic and clinical characteristics of individuals enrolled in this study. Supplemental Table S3. Overview of Ongoing or completed Clinical Trials in HCC Immunotherapy.

## Abbreviations

The following abbreviations are used in this manuscript:

HCC	hepatocellular carcinoma
TME	tumor microenvironment
G0S2	G0/G1 Switch 2
NR	non-responders
R	responders
PBMCs	peripheral blood mononuclear cells
ICB	immune checkpoint blockade
RT-PCR	reverse transcription-polymerase chain reaction
TAMs	tumor-associated macrophages

## References

[1] Llovet J.M., Castet F., Heikenwalder M., Maini M.K., Mazzaferro V., Pinato D.J., Pikarsky E., Zhu A.X., Richard S. (2022). Finn Immunotherapies for hepatocellular carcinoma. Nat. Rev. Clin. Oncol..

[2] Facciorusso A., Tartaglia N., Villani R., Serviddio G., Ramai D., Mohan B.P., Chandan S., El Aziz M.A.A., Evangelista J., Cotsoglou C. (2021). Lenvatinib versus sorafenib as first-line therapy of advanced hepatocellular carcinoma: A systematic review and meta-analysis. Am. J. Transl. Res..

[3] Mandlik D.S., Mandlik S.K., Choudhary H.B. (2023). Immunotherapy for hepatocellular carcinoma: Current status and future perspectives. World J. Gastroenterol..

[4] Liu W.N., Harden S.L., Tan S.L.W., Tan R.J.R., Fong S.Y., Tan S.Y., Liu M., Karnik I., Shuen T.W.H., Toh H.C. (2024). Single-cell RNA sequencing reveals anti-tumor potency of CD56(+) NK cells and CD8(+) T cells in humanized mice via PD-1 and TIGIT co-targeting. Mol. Ther..

[5] Zhou X., Chen Z., Yu Y., Li M., Cao Y., Prochownik E.V., Li Y. (2024). Increases in 4-Acetaminobutyric Acid Generated by Phosphomevalonate Kinase Suppress CD8(+) T Cell Activation and Allow Tumor Immune Escape. Adv. Sci..

[6] Lara S., Akula S., Fu Z., Olsson A.-K., Kleinau S., Hellman L. (2024). The Human Monocyte-A Circulating Sensor of Infection and a Potent and Rapid Inducer of Inflammation. Int. J. Mol. Sci..

[7] Donia M., Svane I.M. (2024). PD-L1 Expression for Tailoring Treatment in Advanced Melanoma-It Is Never That Easy-Reply. JAMA Oncol..

[8] Li X., Liu Y., Gui J., Gan L., Xue J. (2024). Cell Identity and Spatial Distribution of PD-1/PD-L1 Blockade Responders. Adv. Sci..

[9] Cremer J., Brohée L., Dupont L., Lefevre C., Peiffer R., Saarinen A.M., Peulen O., Bindels L., Liu J., Colige A. (2024). Acidosis-induced regulation of adipocyte G0S2 promotes crosstalk between adipocytes and breast cancer cells as well as tumor progression. Cancer Lett..

[10] Qin S., Chen Z., Fang W., Ren Z., Xu R., Ryoo B.-Y., Meng Z., Bai Y., Chen X., Liu X. (2023). Pembrolizumab Versus Placebo as Second-Line Therapy in Patients From Asia With Advanced Hepatocellular Carcinoma: A Randomized, Double-Blind, Phase III Trial. J. Clin. Oncol..

[11] Tu X., Chen L., Zheng Y., Mu C., Zhang Z., Wang F., Ren Y., Duan Y., Zhang H., Tong Z. (2024). S100A9(+)CD14(+) monocytes contribute to anti-PD-1 immunotherapy resistance in advanced hepatocellular carcinoma by attenuating T cell-mediated antitumor function. J. Exp. Clin. Cancer Res..

[12] Pan Y., Shu G., Fu L., Huang K., Zhou X., Gui C., Liu H., Jin X., Chen M., Li P. (2024). EHBP1L1 Drives Immune Evasion in Renal Cell Carcinoma through Binding and Stabilizing JAK1. Adv. Sci..

[13] Ma Y., Zhang M., Yu H., Lu J., Cheng K.K.Y., Zhou J., Chen H., Jia W. (2024). Activation of G0/G1 switch gene 2 by endoplasmic reticulum stress enhances hepatic steatosis. Metabolism.

[14] Mohan D.R., Lerario A.M., Else T., Mukherjee B., Almeida M.Q., Vinco M., Rege J., Mariani B.M.P., Zerbini M.C.N., Mendonca B.B. (2024). Targeted Assessment of G0S2 Methylation Identifies a Rapidly Recurrent, Routinely Fatal Molecular Subtype of Adrenocortical Carcinoma. Clin. Cancer Res..

[15] Lampiasi N. (2024). Macrophage Polarization: Learning to Manage It. Int. J. Mol. Sci..

[16] Li H., Zhou K., Wang K., Cao H., Wu W., Wang Z., Dai Z., Chen S., Peng Y., Xiao G. (2024). A pan-cancer and single-cell sequencing analysis of CD161, a promising onco-immunological biomarker in tumor microenvironment and immunotherapy. Front. Immunol..

[17] Xie Q., Liu X., Liu R., Liang J. (2024). Cellular mechanisms of combining innate immunity activation with PD-1/PD-L1 blockade in treatment of colorectal cancer. Mol. Cancer.

[18] Chen Y., Jia K., Chong X., Jiang L., Peng H., Liu D., Yuan J., Li Y., Feng X., Sun Y. (2024). Implications of PD-L1 expression on the immune microenvironment in HER2-positive gastric cancer. Mol. Cancer.

[19] Chen Z., Soni N., Pinero G., Giotti B., Eddins D.J., Lindblad K.E., Ross J.L., Vallcorba M.P., Joshi T., Angione A. (2024). Monocyte depletion enhances neutrophil influx and proneural to mesenchymal transition in glioblastoma. Nat. Commun..

[20] Yang X., Deng B., Zhao W., Guo Y., Wan Y., Wu Z., Su S., Gu J., Hu X., Feng W. (2025). FABP5(+) lipid-loaded macrophages process tumor-derived unsaturated fatty acid signal to suppress T-cell antitumor immunity. J. Hepatol..

[21] Sun G., Wang Y., Yang L., Zhang Z., Zhao Y., Shen Z., Han X., Du X., Jin H., Li C. (2024). Rebalancing liver-infiltrating CCR3(+) and CD206(+) monocytes improves diet-induced NAFLD. Cell Rep..

[22] Xu Q., Wang Y., Huang W. (2021). Identification of immune-related lncRNA signature for predicting immune checkpoint blockade and prognosis in hepatocellular carcinoma. Int. Immunopharmacol..

[23] Zhou C., Weng J., Liu C., Zhou Q., Chen W., Hsu J.L., Sun J., Atyah M., Xu Y., Shi Y. (2020). High RPS3A expression correlates with low tumor immune cell infiltration and unfavorable prognosis in hepatocellular carcinoma patients. Am. J. Cancer Res..

[24] Facciorusso A., Abd El Aziz M.A., Sacco R. (2019). Efficacy of Regorafenib in Hepatocellular Carcinoma Patients: A Systematic Review and Meta-Analysis. Cancers.

[25] Shen Y., Bai Y. (2025). Effectiveness of regorafenib in second-line therapy for advanced hepatocellular carcinoma: A systematic review and meta-analysis. Medicine.

[26] Zhang X., Coker O.O., Chu E.S., Fu K., Lau C.H., Wang Y.-X., Chan A.W.H., Wei H., Yang X., Sung J.J.Y. (2021). Dietary cholesterol drives fatty liver-associated liver cancer by modulating gut microbiota and metabolites. Gut.

[27] Osna N.A., Rasineni K., Ganesan M., Donohue T.M., Kharbanda K.K. (2022). Pathogenesis of Alcohol-Associated Liver Disease. J. Clin. Exp. Hepatol..

[28] Ganesh S., Kim M.J., Lee J., Zhang Z., Zhao Y., Shen Z., Han X., Ule K., Mahan A., Brown B.D. (2024). RNAi mediated silencing of STAT3/PD-L1 in tumor-associated immune cells induces robust anti-tumor effects in immunotherapy resistant tumors. Mol. Ther..

[29] Xu X., Feng L., Liu Y., Zhou W.X., Ma Y.C., Fei G.J., An N., Li Y., Wu X., Lu X.Y. (2024). Differential gene expression profiling of gastric intraepithelial neoplasia and early-stage adenocarcinoma. World J. Gastroenterol..

