# Supplementary material for: G0S2 Promotes PD-L1 Expression in Monocytes and Influences the Efficacy of PD-1 Inhibitors in Hepatocellular Carcinoma
Source: Genes (Basel). 2025 Apr 13;16(4):448. doi: 10.3390/genes16040448 (PMC12027009; doi:10.3390/genes16040448)
Supplement: Supplementary file 1 [file genes-16-00448-s001.zip › Table S3.pdf]

Supplemental Table S3. Overview of Ongoing or completed Clinical Trials in HCC Immunotherapy

Trial Registration	Study Purpose	Primary Outcome Measures and Secondary Outcome Measures
NCT03062358	Study of Pembrolizumab (MK-3475) or Placebo Given With Best Supportive Care in Asian Participants With Previously Treated Advanced Hepatocellular Carcinoma (MK-3475-394/ KEYNOTE-394)	Overall Survival (OS), Progression Free Survival (PFS), Objective Response Rate (ORR), Duration Of Response (DOR)
NCT06298123	Risk Factor Analysis and Prognostic Modelling of Postoperative Adjuvant Immunotherapy for Hepatocellular Carcinoma	Recurrence-Free Survival (RFS), Overall Survival (OS),
NCT05535998	TACE-HAIC Combined With TKIs and Immunotherapy Versus TACE Alone for Hepatocellular Carcinoma With PVTT	Tumor Response, Overall survival (OS), Progression-free survival (PFS), Conversion rate
NCT05307926	PD-1-based Adjuvant Therapy in High-risk Hepatocellular Carcinoma Patients After Curative Resection	Disease-free survival, Overall Survival, Incidence of Treatment-Emergent Adverse Events [Safety and Tolerability]
NCT05162352	Donafenib Combined with Sintilimab for Advanced HCC: a Single-arm, Single-center, Prospective Study	Progression free survival (PFS), Adverse Events (AEs), Objective response rate (ORR), Disease control rate (DCR)
NCT04767906	Cabozantinib Treatment in a Phase II Study for Patients With Hepatocellular Carcinoma (HCC) Refractory to PD-1 Inhibitors	time on treatment (TT), Overall survival (OS), Progression-free survival (PFS), Duration of response (DoR), Response rates, Median average dose

ClinicalTrials.gov
