# Supplementary figures and images for: G0S2 Promotes PD-L1 Expression in Monocytes and Influences the Efficacy of PD-1 Inhibitors in Hepatocellular Carcinoma
Source: Genes (Basel). 2025 Apr 13;16(4):448. doi: 10.3390/genes16040448 (PMC12027009; doi:10.3390/genes16040448)

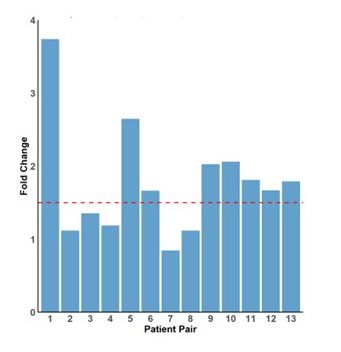

Supplement: Supplementary file 1 [file genes-16-00448-s001.zip › Supplementary Figure S1.jpg]

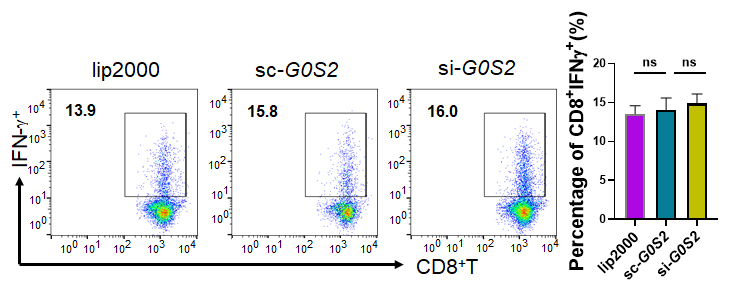

Supplement: Supplementary file 1 [file genes-16-00448-s001.zip › Supplementary Figure S2.jpg]
